# A Remotely Delivered Light-Intensity Physical Activity Intervention for Older Cancer Survivors: Protocol for a Feasibility Randomized Controlled Trial

**DOI:** 10.2196/59504

**Published:** 2024-12-13

**Authors:** Cindy K Blair, Ursa Brown-Glaberman, Scott T Walters, Claire Pestak, Tawny Boyce, Laura Barriga, Ellen Burgess, Bernard Tawfik, Cynthia Killough, Anita Y Kinney, Wendy Demark-Wahnefried, Angela L Meisner, Charles L Wiggins, V Shane Pankratz, Sally Davis

**Affiliations:** 1 Department of Internal Medicine University of New Mexico Albuquerque, NM United States; 2 University of New Mexico Comprehensive Cancer Center Albuquerque, NM United States; 3 School of Public Health University of North Texas Health Science Center Ft. Worth, TX United States; 4 Health Sciences Center University of New Mexico Albuquerque, NM United States; 5 Health Sciences Center – Clinical and Translational Science Center University of New Mexico Albuquerque, NM United States; 6 Department of Biostatistics and Epidemiology School of Public Health Rutgers University Piscataway, NJ United States; 7 Rutgers Cancer Institute Rutgers University New Brunswick, NJ United States; 8 Department of Nutrition Sciences University of Alabama at Birmingham Birmingham, AL United States; 9 O’Neal Comprehensive Cancer Center at the University of Alabama at Birmingham Birmingham, AL United States; 10 Department of Pediatrics University of New Mexico Albuquerque, NM United States; 11 University of New Mexico Prevention Research Center Albuquerque, NM United States

**Keywords:** cancer survivor, physical performance, physical activity, physical function, older adults, activity tracker, mobile phone

## Abstract

**Background:**

Older cancer survivors face age- and treatment-related comorbidities, including physical functional impairment, which are exacerbated by physical inactivity and sedentary behavior. Regular physical activity can reduce this risk, yet less than 30% of older cancer survivors meet the recommended guidelines for physical activity.

**Objective:**

This study aims to describe the design, methods, and rationale for a remotely delivered intervention that uses a whole-of-day approach to physical activity in older cancer survivors. This approach focuses on the accumulation of intermittent bouts of light-intensity activity throughout the entire day by disrupting and reducing sedentary activity. The intervention was guided by social cognitive and self-determination theories and incorporated motivational interviewing.

**Methods:**

The 12-week Move for Your Health trial randomly assigned 64 older cancer survivors to a theory-based physical activity intervention or a waitlist control. A Fitbit (Google) activity tracker and smartphone app were used to promote awareness of activity levels and enable self-monitoring of both activity and inactivity in tandem with health coaching phone calls. Motivational interviewing was used to engage participants and tailor strategies to achieve goals during the 12-week intervention. Data were collected at baseline, immediately after the intervention, and at longer-term follow-up (3 months thereafter). Feasibility outcomes included recruitment, retention, adherence, adverse events, and acceptability. Other outcomes included obtaining the parameter estimates for changes in physical function, physical performance, physical activity, sedentary behavior, and quality of life.

**Results:**

Recruitment for the Move for Your Health randomized controlled trial was completed in June 2023. Data collection was completed in March 2024. Data analyses are ongoing.

**Conclusions:**

The results of this trial will provide information on the feasibility of implementing this intervention in the target patient population, as well as data that will provide information about the potential impact of the intervention on the outcomes. Both of these outcomes will inform the design of a larger randomized controlled trial to more fully test a physical activity intervention in an older cancer survivor population.

**Trial Registration:**

ClinicalTrials.gov NCT05582889; https://clinicaltrials.gov/study/NCT05582889

**International Registered Report Identifier (IRRID):**

DERR1-10.2196/59504

## Introduction

### Background

The unprecedented growth of the number of individuals in the United States who are 65 years and older, combined with the cancer burden in this age group (>55% of diagnoses) and the high survivability of cancer (currently >65%) [1,2], is resulting in a rapidly expanding high-need population of older cancer survivors. Compared with the general population, older cancer survivors are at higher risk for physical function decline and other comorbidities (eg, cardiovascular disease, osteoporosis, fracture, etc), which further exacerbate the risk for functional limitations [3-6]. Given the adverse consequences of functional impairment, including mobility limitations, increased number of falls, hospital or skilled nursing facility admissions, diminished quality of life, premature death, and substantial financial costs [7-10], the importance of implementing strategies to delay or mitigate these effects is crucial. Despite comprising the majority of cancer survivors, few health behavior change interventions are conducted in older survivors with comorbidities [11].

Regular physical activity has been shown to reduce the risk of comorbid conditions [12-14], yet less than 30% of cancer survivors meet the recommended goal (≥150 minutes per week of moderate-intensity physical activity or ≥75 minutes per week of vigorous-intensity physical activity; eg, fast walking or aerobics) [13-17]. Compounding the effects of physical inactivity are the adverse physiologic distinct effects of sedentary behavior [18,19], defined as sitting, lying, or reclining with minimal energy expenditure during waking hours (ie, excludes sleep) [20]. Sedentary behavior is associated with an increased risk of cardiovascular disease [21-24], premature mortality [25,26], and decreased physical function [27,28], even among individuals who meet recommended activity guidelines.

The predominant focus in physical activity research has been on moderate- to vigorous-intensity physical activity (MVPA) since this intensity level is associated with the greatest health benefits [12]. However, older cancer survivors with functional impairment may have different needs, preferences, and barriers to being physically active and thus may require a different approach. Light-intensity physical activity (LPA), such as leisurely walking, is associated with better physical health [29-34], including physical function [35-39] and emotional well-being [29,33,34,40], independent of MVPA. These associations may only be apparent or appear stronger in adults who are older, less physically active, or with impaired lower extremity function [25,30,36,41,42]; that is, the effect sizes are larger or only reach statistical significance when stratified by these categories. Changing the focus from MVPA to LPA may represent a more achievable and sustainable target for vulnerable populations such as older survivors with comorbidities. Despite evidence of the health benefits of LPA, few randomized controlled trials (RCTs) have been conducted to evaluate the effects of LPA on health outcomes.

The majority of physical activity studies have been clinic or center based, focused on common cancers (breast, prostate, and colorectal), and targeted behavior (ie, physical activity) as the primary outcome [43,44]. Few studies have focused on medically underserved survivor populations (older adults, racial and ethnic minority groups, and rural dwellers) and targeted outcomes meaningful to survivors (eg, patient-reported outcomes) [43]. Thus, there is a critical need for lifestyle activity interventions that are effective, low-cost, and accessible to older cancer survivors from diverse backgrounds, designed to be feasible, relevant, and meaningful for them. A distance- and technology-based approach (in both intervention delivery and outcomes assessment) would expand the reach of interventions to underserved and underrepresented minority survivor populations.

Consumer wearable activity trackers are a low-cost, easily accessible, and potentially scalable strategy to promote behavior change and facilitate remote intervention delivery. Many trackers, along with their phone applications, use multiple behavior change techniques associated with improving physical activity, such as incremental goal setting, review of behavioral goals, self-monitoring of behavior, and feedback on behavior. These devices can measure progress, provide feedback in real-time, and even deliver prompts or cues to action. Several well-designed pilot or feasibility studies have evaluated wearable activity trackers as an intervention tool to increase physical activity [45-49], interrupt and replace sedentary behavior [50,51], or both [52-54]. These studies have primarily enrolled breast, prostate, or colorectal cancer survivors and focused on brisk walking as a form of MVPA. In contrast, there have been few studies using trackers that have also focused on LPA as the prescription.

A challenge of distance-based trials is the availability of low-resource assessments of objective data, such as physical performance (eg, the Timed Up and Go test and the chair stand test [55-57]). Before the COVID-19 pandemic, physical performance testing for objective measurement of physical functioning was primarily assessed in person, which may have excluded rural, older, or other individuals unwilling or unable to travel. Low-cost, valid, and reliable methods to remotely conduct such testing in the home setting were limited. We designed a study to evaluate the validity, reliability, acceptability, and, most importantly, safety of using videoconferencing to remotely assess tests of functional mobility and strength among older cancer survivors in their own homes [58]. The COVID-19 pandemic has accelerated the use of videoconferencing to remotely deliver physical performance testing [59-61], potentially expanding the reach, scalability, and dissemination of interventions to cancer survivors who may not otherwise have access, especially those residing in rural areas [58].

### Objectives

To address these research gaps, we designed the Move for Your Health (MY Health) study, a 12-week RCT to determine the feasibility of using a whole-of-day approach to physical activity to improve physical function among older cancer survivors. The whole-of-day approach focuses on the accumulation of intermittent, short bouts of activity (stepping) throughout the entire day by disrupting sedentary activity. The specific aims are to (1) determine the feasibility and acceptability of a remotely delivered lifestyle activity intervention by assessing recruitment, retention, adherence rates, and participant satisfaction and monitoring adverse events; (2) estimate effect sizes for primary and secondary end points for the adoption (baseline to after the intervention) and maintenance (3 months after the intervention) of the intervention; and (3) conduct a process evaluation using mailed surveys and intervention fidelity assessments to inform a larger definitive trial with broader dissemination.

## Methods

### Study Design and Setting

**Figure 1:**
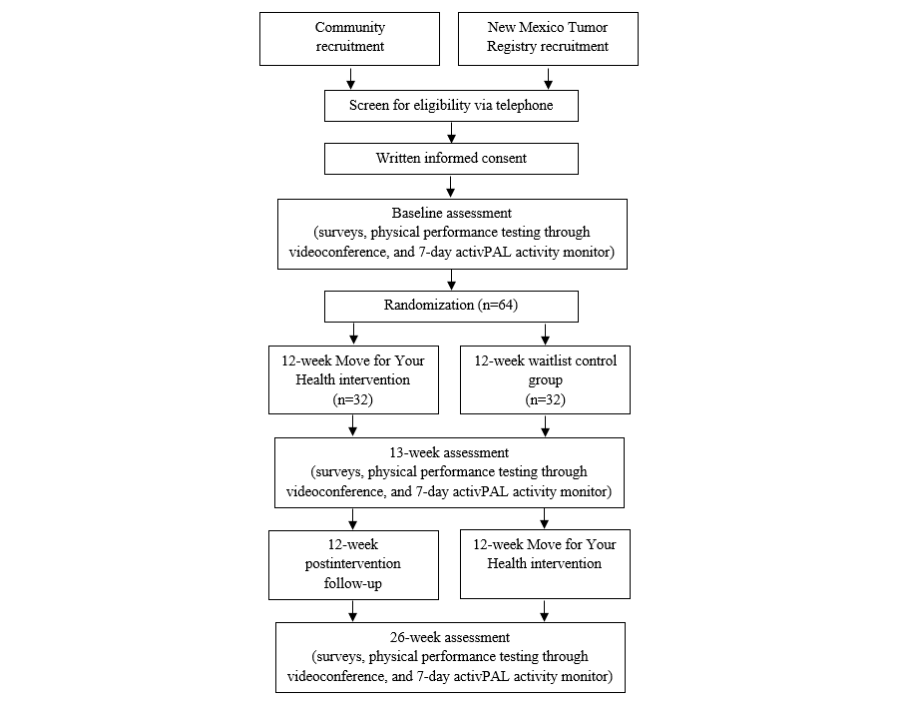
Study flow diagram.

MY Health is a waitlist RCT that examined the feasibility of an intervention to promote LPA and disrupt sedentary activity using a whole-of-day approach in 64 older cancer survivors. Data collection occurred at baseline, immediately after the intervention, and at longer-term follow-up (3 months after the intervention; Figure 1). Both intervention delivery and data collection were conducted remotely in participants’ homes.

### Recruitment and Eligibility

A multipronged recruitment approach was used, including (1) identification of cancer survivors from the New Mexico Tumor Registry (NMTR), one of the founding registries of the National Cancer Institute’s Surveillance Epidemiology and End Results (SEER) Program [62,63]; (2) clinician referrals; and (3) flyers distributed to cancer survivor support groups, community and senior centers, and health fairs and other community events with the assistance of a community engagement liaison specialist from the University of New Mexico Health Sciences Center. Random quota sampling based on ethnicity (Hispanic vs non-Hispanic) and geography (rural vs urban) was used to enhance balance across key subgroups. The sample frame included the entire state of New Mexico. Rural status was determined based on the population size (<50,000=rural; ≥50,000=urban) of the participant’s place of residence. This definition incorporated the population size of the town or city from the Rural-Urban Commuting Area classification system [64] but ignored the commuting area codes to better reflect the participants’ immediate environment, since this is a home-based study among older adults (ie, to support physical activity near one’s home). Eligible individuals identified by the NMTR were mailed a letter that briefly introduced the study and provided recipients an opportunity to refuse to participate. Following a defined time period, contact information was provided to the study team. Individuals were then mailed a letter explaining the study and inviting their participation; 2 consent forms (one to keep and one to mail back) were included with the letter. Individuals (identified by the NMTR) not refusing contact, who were referred by their provider, or who self-referred to the study were contacted by phone to discuss the study, verify eligibility, and begin the consent process; a maximum of 4 calls were made. The total time to accrue the full sample, contact and cooperation rates, and reasons for nonparticipation were tracked as part of the feasibility assessment of recruitment.

Eligibility criteria included (1) age 65 years or older; (2) residing in New Mexico and not planning to move out of state during the next year; (3) previous cancer diagnosis and completed primary treatment (patients with metastatic cancer were eligible with physician approval); (4) mild-to-moderate physical function impairment (raw score between 15 and 29 on the 8-item Patient-Reported Outcomes Measurement Information System physical function survey; representing approximately 0.5 and 1.5 SD below the population mean, respectively); (5) able to speak, read, and understand English or Spanish; (6) participating in less than 120 minutes per week of MVPA (allows for self-report bias in overreporting physical activity or misclassification of light-intensity activity as moderate-intensity activity, determined using the Godin Leisure-Time Exercise Questionnaire—low respondent burden); (7) living independently and capable of walking 3 blocks (approximately 1300 steps or 0.25 mile) without an assistive device (eg, cane or walker) and without stopping to rest; (8) own a smartphone, or be willing to use a study-provided smartphone, capable of running the Fitbit app (Google) and accessing the internet; and (9) willingness to be randomized to either study arm and to wear an activity tracker.

Exclusion criteria included (1) severe impairments (in seeing or hearing) or preexisting medical limitations that would constrain engaging in daily LPA (eg, severe orthopedic conditions, pending hip or knee replacement, dementia, oxygen dependent, or chronic vertigo) or (2) currently participating in a program (eg, personal or structured program) to decrease sedentary time or increase physical activity.

### Randomization

Upon completion of the baseline assessment, participants were block-randomized with equal allocation to 2 arms (intervention vs waitlist control) within 4 strata defined by self-reported ethnicity (Hispanic vs non-Hispanic) and geographic location (urban vs rural). A computer-generated allocation table (created by a statistician) was uploaded into the Research Electronic Data Capture (REDCap; Vanderbilt University) database, and allocation assignment was announced by the project manager upon completion of the baseline assessment. Given the trial design, it was not possible to blind the participants or the health coaches to the group allocation, though study statisticians were blinded.

### MY Health Intervention

#### Conceptual Framework

**Figure 2:**
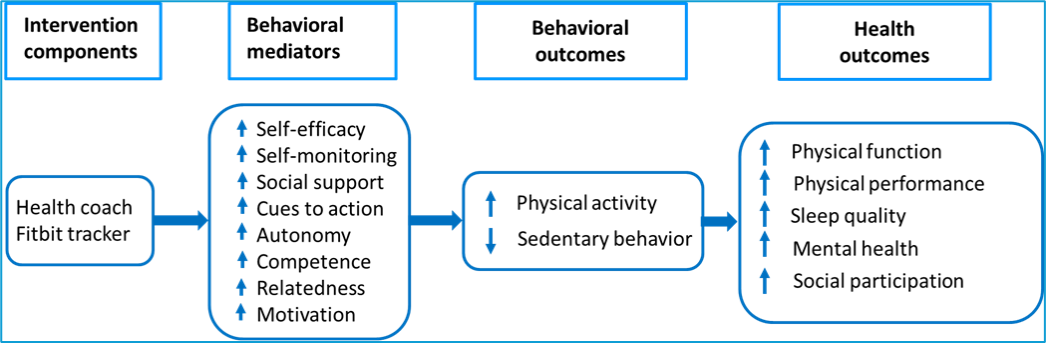
The Move for Your Health (MY Health) study conceptual model.

The remotely delivered lifestyle activity intervention was guided by social cognitive theory and self-determination theory and used motivational interviewing (MI; Figure 2). Social cognitive theory emphasizes self-efficacy, behavioral capability (knowledge and skills to perform a behavior), self-monitoring, and outcome expectancies to promote behavior change [65,66]. Self-determination theory posits that intrinsic versus extrinsic motivation leads to a greater commitment to behavior change and maintenance of change [67]. Intrinsic motivation is more likely when 3 core psychological needs are met: autonomy, competence, and relatedness [67,68]. MI is a person-centered method of communication to help resolve ambivalence related to behavior change, enhance intrinsic motivation to change, and brainstorm ways to overcome barriers [69].

#### Overview and Goals

The goal was to encourage participants to be more active throughout the day—a whole-of-day approach—without regard for minimum intensity or duration of activity bouts. Walking, important for maintaining mobility and independence, served as the primary form of LPA. The primary behavioral target was to reach and maintain an extra 3000 steps per day above baseline (established during the second week post enrollment). This target represents approximately 30 extra minutes of LPA [70] and is associated with health benefits [29,71]. The secondary behavioral target was to increase the number of active h/d (defined as >250 steps/h in Fitbit) to at least 10 h/d to encourage activity throughout the day and to interrupt and replace sedentary time. Frequent interruption of sitting is associated with better physical function [36,72-74]. To promote gradual and sustained change, participants were asked to increase the number of steps per day during weeks 3-12 (Table 1). Participants were asked to “practice” achieving their new step goal on at least 3 days during the first week and then at least 5 days during the second week. This allowed participants to have one or more low-activity days and still be able to achieve their step goal. In accordance with self-determination theory and MI, participants chose their own strategies for becoming more active and achieving their weekly goals with guidance and (technology) support from their health coach.

**Table 1:** Intervention schedule.

Week	Study activity	Active hours per day^a^, n	Targeted steps per day (above baseline), n	Minimum days meeting step goal, n
0-1	Baseline assessment, randomization, and Fitbit mailed to the participant	—^b^	—	—
2	Establish Fitbit baseline step data	—	—	—
3	Health coaching call	6	1000	3
4	—	6	1000	5
5	Health coaching call	7	1500	3
6	—	7	1500	5
7	Health coaching call	8	2000	3
8	—	8	2000	5
9	Health coaching call	9	2500	3
10	—	9	2500	5
11	Health coaching call	10	3000	3
12	—	10	3000	5
13	Postintervention assessment	—	—	—
14-25	Maintain goals (no study contact)	—	—	—
26	3-month postintervention assessment	—	—	—

^a^An active hour is defined by Fitbit as 250 or more steps during an hour (awake hours only).

^b^Not applicable.

#### Fitbit Inspire 3 Wearable Activity Tracker and App

A Fitbit activity tracker and mobile app were used to promote awareness and enable self-monitoring of both activity (steps per day) and inactivity (activity tracker vibrates as a reminder to move each hour). During week 2, participants in the intervention group were mailed the Fitbit Inspire 3 (Google) activity tracker, with instructions for installing the app on their smartphone and for using the Fitbit app. The distinguishing feature of this tracker is an activity reminder, in which the tracker gently vibrates if fewer than 250 steps were taken in the previous 50 minutes and provides a suggestion to take more steps in the next 10 minutes (to achieve a goal of ≥250 steps/hour). Data from the tracker were wirelessly synced to the app. Participants were instructed to wear the tracker during waking hours and were encouraged to check their activity by their app at least once a day. The app includes a daily summary of the total steps and number of active hours (hours with ≥250 steps). The health coach assisted with the installation, reviewing instructions for using the tracker and app, setting goals (steps per day, number of active hours), and checking their progress. If needed, a family member or close friend was included in telephone calls to assist the participant.

#### Health Coaching Calls

Bilingual research assistants were trained in MI core processes (engaging, focusing, evoking, and planning) and microskills (open-ended questions, affirmations, reflections, and summaries) to promote behavior change and use in their role as health coaches. MI techniques and microskills were used to guide 5 sessions of telephone-based health coaching. While these calls were designed to occur during weeks 3, 5, 7, 9, and 11 (45 minutes for initial 1-2 calls, then 15-20 minutes each), participants were allowed to reschedule due to illness, etc. Key intervention topics and techniques included establishing rapport; eliciting knowledge about the health benefits of physical activity and interrupting sedentary time; assessing motivation, confidence, and perceived importance and barriers to becoming more active; eliciting change talk; and enhancing participants’ self-efficacy (competence) and motivation to be more active (eg, small, achievable goals). The health coach also provided tech support to the participants for setting up their Fitbit and using their mobile app. The health coaches regularly checked Fitabase (Small Steps Labs LLC) and sent reminders (telephone, email, or text, depending on the participant’s preference) if participants had not synced their Fitbit in 4 or more days or were not wearing their Fitbit (limited or no steps recorded).

#### Visual Aids for Health Coaching Calls

Participants were mailed visual aids to serve as a reference for discussions with the health coach. The health coaches followed a semistructured guide that corresponded to the visual aids. The initial call included a discussion about the number of daily steps associated with good health in older adults (7000-9000) [75-77] and the benefits of spreading out activity throughout the day (ie, breaking up sedentary time through at least minimal stepping each hour). Participants were asked to rate the importance of meeting the recommended level of daily steps and to provide reasons why it was important for them to be physically active; examples for participants to consider include reducing stress and anxiety, increasing energy levels, enhancing balance, improving sleep quality, etc. After reviewing the baseline Fitbit data, participants were provided with the suggested goals (daily steps and active hours) for the next 2 weeks (Table 1) and examples for adding 1000 steps per day (one 10-minute or two 5-minute brisk walk, one 15-minute leisurely walk, or adding at least an extra 150 steps for 6 hours). The participants identified strategies and created a plan for achieving these goals. Examples for participants to consider included playing music and dancing, walking the dog, parking further from an entrance, etc. While examples were provided, participants were encouraged to choose their own strategies. The calls ended with a review of how to set the step goal and the reminders to move in the Fitbit app. Subsequent calls reviewed the previous 2 weeks and created plans for meeting subsequent goals. For example, participants who achieved their step goal during the previous week might have chosen the study recommended goal (increase by 500 steps), whereas participants who did not meet the goal (eg, due to illness) might have chosen to maintain their previous step goal. Participants may have kept their same strategies or identified new strategies for achieving their goals and then created a plan for the next 2 weeks. Table 2 provides examples of the use of MI and visual aids by the health coaches in the theoretically-guided approach.

**Table 2:** Examples of how behavioral theory and motivational interviewing (MI) were used to promote physical activity among older cancer survivors.

Theoretical construct	Health coaching with MI and visual aids
Autonomy support	Visual aids include examples of strategies to increase steps; however, participants select strategies that work best for them. During subsequent health coaching calls, participants decide whether to maintain their step goal or increase it and by how much. The health coach actively emphasizes the individual’s own personal control and choice in decisions.
Motivation	MI is a person-centered method of communication for helping to resolve ambivalence around behavior change and enhance an individual’s intrinsic motivation to change. Each health coaching call uses MI core processes (engaging, focusing, evoking, and planning) and micro skills (open-ended questions, affirmations, reflections, and summaries) to promote behavior change. For example, the health coaches may call attention to positive progress or evidence of strength and competence (affirmations). Reflections and summaries can be used to focus on the most important reasons for change and the plan of action, thereby facilitating a transition from talk about motivation to talk about action.
Outcome expectations	A visual aid slide during the first health coaching call includes an importance ruler: How important is it to you to be meeting the recommended level of physical activity (scale of 1 to 10; not important to very important)? The follow-up question encourages change talk (versus sustain talk): Why did you say that number and not a lower number?
Self-efficacy and competence	A visual aid slide during each health coaching call includes a confidence ruler: How confident are you that you can meet these goals during the next 2 weeks (scale of 1 to 10; not confident to very confident)? The follow-up question encourages change talk (versus sustain talk): Why did you say that number and not a lower number? The health coach encourages incremental and achievable goals.
Self-monitoring	Participants were taught how to use the Fitbit app to review their daily and weekly progress regarding their step goal and their number of active hours.
Social support and relatedness	Each health coach is assigned participants and provides support and encouragement to their participants throughout the study. The health coach will provide suggestions when asked. In addition, the visual aids include examples of strategies to include other people in physical activities.

#### Intervention Fidelity

We used evidence-based MI training procedures identified in previous studies [78,79]. The health coaches received an initial 1.5 days (12 hours) of in-person MI training by an experienced MI trainer (STW). Before interacting with participants, the coach completed 4 practice sessions and received feedback through 4 phone supervision sessions. These sessions were spread over 6-8 weeks to allow for further review of materials between feedback sessions. STW coded the sessions for MI fidelity by using the MI Treatment Integrity Coding System (version 4.1) [80] and used this information during supervision sessions. During the study period (15 months), each health coach submitted 1 audio recording and participated in 1 phone supervision per month (months 1-3), then every other month (months 4-15). Health coaches were provided with training on the intervention design, measures, and outcomes. Fidelity to the intervention delivery was ensured through the use of the operations manual, spreadsheet software for tracking day-to-day activities, and weekly team meetings.

#### Waitlist Control

A waitlist control was selected to help boost retention [81] and balance validity, cost, and time. Participants were asked to maintain their usual lifestyle during the initial 12 weeks. Upon completion of the 13-week assessment, waitlisted participants were mailed a Fitbit Inspire 3, instructions, and visual aids and received the full intervention (Table 1).

#### Retention

To compensate participants for their time, a US $50 merchandise card was provided for each outcome assessment (US $150 total). In addition, both groups were permitted to keep the Fitbit activity tracker after the study ended to maintain activity (at the time of the intervention, a US $100 value).

#### Data Collection

Data were collected by mailed packets (activity monitor and questionnaires) and videoconferencing (physical performance tests) at baseline, immediately after the intervention, and at longer-term follow-up (3 months after intervention). The assessment packet included an activity monitor (for objective measurement of steps, LPA, and sedentary behavior), questionnaires, and print instructions for attaching the activity monitor and for completing the remote assessment of physical function. In addition, participants were emailed links to the YouTube videos of the instructions. Participants were instructed to wear the activPAL (PAL Technologies Ltd; using 24-hour protocol) activity monitors [82] for 1 week, consistent with other studies [40,83,84]. At the end of the week, participants completed questionnaires on both sedentary behavior and physical activity to provide context to the objective data. Instructions were provided for returning (through self-addressed stamped mailer) the monitor and questionnaires to study staff. The health coach called to verify receipt of the assessment packet, review the instructions, if necessary, answer any questions, and schedule the remote assessment of physical performance. Participants were provided an option to complete the study surveys online through a REDCap database. Study data were collected and managed using REDCap electronic data capture tools hosted at the University of New Mexico [85]. REDCap is a secure, web-based application designed to support data capture for research studies, providing (1) an intuitive interface for validated data entry, (2) audit trails for tracking data manipulation and export procedures, (3) automated export procedures for seamless data downloads to common statistical packages, and (4) procedures for importing data from external sources [85].

Fitabase (Small Steps Labs LLC) was used to collect data from participants’ Fitbit trackers. The data collected by Fitabase included total daily steps, number of steps per minute, estimated energy expenditure, distance moved, minutes of vigorous, moderate, and light activity, minutes of sedentary time, sleep length and quality (if Fitbit worn overnight), heart rate, and manually entered and automatically detected physical activities such as walking or running. Fitabase also indicated the last day and time each study Fitbit tracker was synced. Each study participant was deidentified with a participant ID.

Based on our earlier work [58], physical performance tests were conducted in the home setting and remotely assessed using videoconferencing software. Participants used their smartphone, tablet, or camera-enabled computer for the remote assessment. If a participant did not have a suitable device, the study provided a smartphone to capture the physical performance tests. Both written and video instructions were provided that demonstrated the performance tests, safety measures, and how to prepare for the videoconference session with the study investigator. The investigator (TB) performed the assessment from a remote location using videoconferencing software (Zoom [Zoom Communications, Inc] [86] or Skype [Microsoft] [87]; participant’s choice). During the videoconference, the investigator reviewed the instructions for the tests and timed the tests. A separate software (Snagit [TechSmith]) run on a study computer was used to record the assessments for later viewing for quality control purposes.

#### Feasibility and Acceptability Outcomes

Although primary enrollment and data capture activities have been completed, final analyses have not yet been completed. For this feasibility study, our goal was to meet the accrual target of 64 inactive, older cancer survivors within 18 months, retain 80% of the sample, and achieve 80% adherence and fidelity to the intervention while minimizing the number of adverse events. Retention will be calculated as the percentage of participants who completed the final assessment. Adherence to the intervention will be calculated as (1) the percentage of participants who complete all 5 health coaching calls and (2) the percentage of days that participants wore their Fitbit during the intervention. Fidelity to intervention delivery will be calculated as the average score on global ratings for MI (scale of 1 to 5; higher scores are better), which includes cultivating change talk, softening sustain talk, partnership, and empathy. The number of adverse events that were attributable, possibly attributable, or not attributable to the intervention were tracked and reported. Satisfaction with the intervention will be evaluated by a 13-item survey that we developed to assess the level of agreement or disagreement (5-point Likert scale, ranging from strongly disagree=1 to strongly agree=5) on questions about the Fitbit activity tracker, the Fitbit app, and the health coach. Example questions include “The Fitbit activity tracker made me more aware of my activity and inactivity during the week,” “The Fitbit mobile phone app was easy to use,” and “Talking to a Health Coach helped me to identify strategies to be more physically active (get more steps).” Satisfaction with the intervention will be calculated as the average score.

#### Primary and Secondary Outcomes

The measures and a brief description for each primary outcome, secondary outcome, safety assessments, covariates, and theoretical constructs are provided in Table 3.

**Table 3:** Outcomes and measures (T0=week 0 (before randomization); T1=week 13; and T2=week 26).

Outcome, measure, and description			T0		T1		T2
**Primary outcome**							
	Physical function	PROMIS^a^ physical function short form (8a): 8 items to assess the ability to perform daily activities ranging from basic care to vigorous activities [88,89].	✓	✓		✓	
**Secondary outcomes**							
	**Physical performance**						
		A total of 2 standard gerontology assessment tests were used to measure physical performance. Both of these tests are included in the CDC^b^ STEADI^c^ toolkit for the assessment of falls [55].					
		A 30-second chair stand test: number of times a person comes to a full standing position from a chair in 30 seconds; a measure of lower extremity strength and dynamic balance.	✓	✓		✓	
		A 4-stage balance test: includes standing in each of four positions for 10 seconds: (1) feet side-by-side, (2) semitandem stand (1 foot slightly behind but touching the other foot), (3) tandem stand (1 foot in front of the other, heal touching toe), and (4) stand on 1 foot.	✓	✓		✓	
	**Physical activity and sedentary behavior**						
		Objective measures: Daily LPA^d^, MVPA^e^, and sedentary behavior were objectively measured before and after the intervention using the activPAL monitor [90]. The activPAL, worn on the thigh, includes both a triaxial accelerometer and an inclinometer (to detect change in posture, ie, sitting vs upright posture) [91-94].	✓	✓		✓	
		Operational definitions [91]: (1) Total LPA and MVPA: sum of stepping bouts with a step rate of <100 and ≥100 steps per minute, respectively; (2) total sedentary behavior: sum of all sitting or lying bouts; (3) total steps per day; and (4) total waking hours with ≥250 steps [70,95].					
		Subjective measures: (1) The Godin Leisure-Time Exercise Questionnaire [96,97] provided context for activities that changed in frequency, intensity, and duration during the intervention as objectively measured by activPAL, and (2) the Sedentary Behavior Questionnaire [98] was used to estimate self-reported sedentary activities during a typical weekday and a typical weekend. Response items range from none to 6 or more hours per day for 9 common activities (eg, watching television, using a computer, etc).	✓	✓		✓	
	**Quality of life**						
		PROMIS-57 Profile [88]: a combination of short forms designed to assess patient-reported outcomes across a variety of chronic diseases, including cancer [99-102]. It includes 8 items from 7 domains (anxiety, depression, pain interference, fatigue, sleep disturbance, satisfaction with participation in social roles, and physical function) using a 5-point Likert-type scale; 1 item for pain intensity (an 11-point rating scale). Scores are normed to a general population [103].	✓	✓		✓	
**Safety assessment**		Measured by surveys, participants were encouraged to contact the study team immediately for events that occurred between time points.	✓	✓		✓	
	Fall history	Defined as “unintentionally coming to rest on the ground, floor, or other lower level” [104]. The number of falls, nature of falls, and resulting injuries during the past 6 months were recorded before the remote physical performance assessment to inform safety measures, for example, not safe to perform tests, 2 (instead of 1) people needed to supervise or provide support.	✓	✓		✓	
	Fall self-efficacy	FES-I^f^: self-reported level of concern about falling either inside or outside the home for various social and physical activities (16 questions) [105,106].	✓	✓		✓	
**Covariates**		Used to characterize the study population or as predictors of adherence or moderators of intervention effects.					
	Sociodemographics	Age, sex, race and ethnicity, education, income range, marital status (self-report)	✓				
	Health-related behaviors	Smoking status; height and weight to calculate BMI (kg/m^2^) (self-report)	✓				
	Tech-savviness	A brief survey about the use of and comfort level of using their smartphone (developed for this study). Used to prepare study team members to support study participants through the technological aspects of the study (setting up and using Fitbit and app, videoconferencing).	✓				
	Cancer data	Obtained from the New Mexico Tumor Registry (cancer site, stage, age, and year of diagnosis, and summary treatment) and self-report (treatment (yes/no): surgery, chemotherapy, radiation, hormone therapy; year primary therapy completed)	✓				
	Comorbidities	OARS^g^ Comorbidity Index: assesses the number of chronic medical conditions (n=35) and symptoms (n=8) and whether each condition/symptom interferes with activities (not at all, a little, or a great deal) [107].	✓	✓		✓	
**Theoretical constructs**							
	Self-efficacy	Domain specific; we measured confidence for using a Fitbit tracker and app, and increasing the number of active hours and steps.	✓	✓		✓	
	Self-monitoring	Fitbit data will be used to evaluate self-monitoring, for example, the percentage of days the Fitbit tracker was worn and the percentage of days within 5% of reaching the daily step goal.	✓	✓		✓	
	Social support	Social Support for Exercise Survey: 10 items using a 5-point scale to measure support from family and friends while making changes to physical activity behavior [108].	✓	✓		✓	
	Outcome expectations	Multidimensional Outcome Expectations for Exercise Scale: 15 items to assess physical, social, and self-evaluative beliefs about the consequences of being physically active [109,110].	✓	✓		✓	
	Motivation	BREQ-2^h^: 19 items to assess autonomous motivation on a continuum from amotivation to intrinsic motivation. Was adapted using identical anchors for LPA [111,112].	✓	✓		✓	
	Autonomy	Perceived Autonomy Support—Sport Climate Questionnaire: 15 items to assess autonomy support of the health coach to characterize the quality of the social environment for influencing motivation [113].	✓	✓		✓	

^a^PROMIS: Patient-Reported Outcomes Measurement Information System.

^b^CDC: Centers for Disease Control and Prevention.

^c^STEADI: Stopping Elderly Accidents, Deaths, & Injuries.

^d^LPA: light-intensity physical activity.

^e^MVPA: moderate- to vigorous-intensity physical activity.

^f^FES-I: Falls Efficacy Scale-International.

^g^OARS: Older Americans Resources & Services.

^h^BREQ-2: Behavioral Regulations in Exercise Questionnaire.

### Statistical Analysis

#### Power

Using a 2-sided, 2-sample *t* test, with a significance level of .05 and 25 people per arm, we estimated that we would have 80% power to detect a mean difference of 8.1 (SD 10) in physical function scores (a large effect size). To allow for 20% attrition, we planned to enroll 32 participants per arm. This feasibility trial will provide estimates of within-group SDs and between-group differences of physical function scores for the target population. These results will inform the design (sample size, effect size) of a larger trial powered to examine the efficacy of the intervention.

#### Analysis Overview

Outcome variables violating the normality assumption will be transformed before analyses. The pattern of missing data will be evaluated [114], and the appropriate procedures for multiple imputation will be used. Primary analyses will be performed according to the intent-to-treat principle; as this is a feasibility study, complete case analyses will also be explored. activPAL data will be downloaded using PAL technologies software (version 8) [115] and analyzed using SAS (version 9.4, SAS Institute) and R software (version 3.4.3; R Foundation for Statistical Computing). Raw and event data (start and stop time for sitting or lying, standing, and stepping) will be processed using standard methods, for example, determining valid wear time [116-118], threshold values for activity intensity [119,120], etc. A valid day is defined as 10 or more hours of wear during awake time. Consistent with the intention-to-treat principle and similar to other recent trials [53,54,121], participants with at least one valid day of activPAL data at baseline will be included in the analyses. If needed, a sensitivity analysis will be conducted that will exclude participants with fewer than four valid days of activPAL data. Quality controls (daily logs and visual examination of heat maps) will be used to check the classification of accelerometer data. Accelerometer methods will be published according to guidelines [122-124].

#### Preliminary Estimates of Effect Sizes

We hypothesize that physical function scores will improve in the intervention group compared with the control group. We will calculate means and precision estimates for between-arm differences in physical function scores at each time point. A linear mixed-effects model will be used with group, time, and group-by-time interaction, adjusted for ethnicity, rural or urban status, and baseline values as needed. The appropriate variance-covariance structure will be selected to account for the potential correlation among repeated measurements from the same participant. The primary test of interest is the group-by-time interaction, indicating whether there was a difference between groups in the primary outcome over time. Similar analyses will be conducted for secondary outcomes.

#### Process Evaluation

Acceptability and satisfaction related to the intervention will be evaluated by a mailed survey. Process data and other study-related outcomes will be reviewed to identify the next steps and strategies to ensure the most appropriate design and methods for the future, larger RCT, with a potential for broad dissemination and implementation.

### Ethical Considerations

The study protocol and materials were approved by the University of New Mexico Health Sciences Center Institutional Review Board (17-335). Written informed consent was obtained from all study participants. The trial was registered on ClinicalTrials.gov (NCT05582889).

## Results

Enrollment began in October of 2022 and ended in June of 2023. The study enrolled and randomized the targeted number of 64 participants: 40% rural and 33% Hispanic. Data collection was completed in March of 2024. Data analyses for the primary and secondary outcomes are expected to be completed by December 2024. The results of these analyses will be reported in a separate manuscript.

## Discussion

### Expected Findings

Despite decades of research on physical activity, there are few interventions that are effective and accessible to older cancer survivors with comorbidities and functional impairment. Most studies have focused on MVPA, which is difficult for some older survivors to adopt and especially to maintain [125]. Also, academic center– or clinic-based interventions have limited potential for dissemination. Evidence suggests that LPA also confers physical and psychosocial health benefits [29,33,35,126,127], while sedentary activity is associated with deleterious effects [21-24,28,128]. Thus, we proposed a remotely delivered intervention focused on increasing LPA and reducing sedentary behavior throughout the day—likely more obtainable goals—for maintaining and improving physical function in vulnerable populations. By reducing barriers to participation, a remote delivery and assessment approach can expand the reach to underserved cancer survivors who are older or from rural areas.

One of the limitations of this study is that it only included a 3-month follow-up of activity. A future study to examine efficacy in a fully powered trial will include a longer follow-up period. Although our goal was to enroll 50% Hispanic and 50% rural cancer survivors, our actual enrollment was 33% Hispanic and 40% rural. More time and resources would have been needed to achieve our original goal. While heterogeneity exists between cancer types, the common features targeted by this intervention are physical functional impairment and a physically inactive lifestyle, which are not cancer-type specific. This broad-based sample of older cancer survivors improves generalizability and potential for widespread dissemination. By design, this study is focused on promoting LPA, primarily through walking, as opposed to the currently recommended guidelines of 150 minutes per week of moderate-intensity physical activity (or 75 minutes per week of vigorous-intensity physical activity) plus 2 days of resistance training. However, even the guidelines mention “some activity is better than none.” Our proposed approach could lay the foundation for further increases in activity intensity or duration with the potential to achieve even greater health benefits.

### Conclusion

The proposed intervention is responsive to the call for studies that include more diverse cancer survivor populations [11,43], rigorously designed trials to evaluate the benefits of increasing LPA and reducing sedentary behavior in older adults [129-131], and interventions with the potential for dissemination and implementation in a variety of settings [11]. The results of this trial will provide information on the feasibility of implementing this intervention in the target patient population, as well as data that will provide information about the potential impact of the intervention on the outcomes. Both of these outcomes will inform the design of a larger RCT to fully test a physical activity intervention in an older cancer survivor population. Rigorously designed trials such as this will help elucidate the amount of LPA and reduction in sedentary behavior needed to improve physical functioning and other health outcomes in older cancer survivors with comorbidities.

## Abbreviations

LPA: light-intensity physical activity
MI: motivational interviewing
MVPA: moderate- to vigorous-intensity physical activity
MY Health: Move for Your Health
NMTR: New Mexico Tumor Registry
RCT: randomized controlled trial
SEER: Surveillance Epidemiology and End Results

## Data Availability

Deidentified data will be made available from CKB upon reasonable request. Data to be provided will be group-level data unless participant-level data can be released without revealing an individual’s identity. Please contact CKB at CiBlair@salud.unm.edu. Data will not be available until the primary and secondary results have been published.
